# Improving Access to HIV Prevention Services in Community Pharmacies in the US Southeast: Protocol for a Hybrid Type 1 Effectiveness-Implementation Study

**DOI:** 10.2196/72283

**Published:** 2025-12-03

**Authors:** Daniel I Alohan, Alexis Hudson, Chante Hamilton, Christina Chandra, Seth Zissette, Rachel Rothman, Sophia A Hussen, Alvan Quamina, Robert H Lyles, Jessica M Sales, Claire E Sterk, Henry N Young, Natalie D Crawford

**Affiliations:** 1 Department of Behavioral, Social, and Health Education Sciences Rollins School of Public Health Emory University Atlanta, GA United States; 2 Department of Epidemiology Rollins School of Public Health Emory University Atlanta, GA United States; 3 Georgia Commission on Family Violence Atlanta, GA United States; 4 Hubert Department of Global Health Rollins School of Public Health Emory University Atlanta, GA United States; 5 NAESM, Inc Atlanta, GA United States; 6 Department of Biostatistics and Bioinformatics Rollins School of Public Health Emory University Atlanta, GA United States; 7 Department of Clinical and Administrative Pharmacy College of Pharmacy University of Georgia Athen, GA United States

**Keywords:** HIV prevention, PrEP, HIV, implementation science, protocol, HIV testing, PrEP screening, prophylaxis, access, pharmacy, pre-exposure prophylaxis

## Abstract

**Background:**

Despite advancements in HIV prevention, many Americans, particularly those from historically underserved communities (eg, racially and sexually minoritized individuals and people who use drugs), continue to face significant barriers to accessing crucial HIV prevention services such as HIV testing and pre-exposure prophylaxis. Integrating these services into community pharmacies is a viable yet underused solution to overcoming access-related challenges. However, few studies have used an implementation science approach to assess the implementation and effectiveness of such services in pharmacy settings.

**Objective:**

The Pharmacy-Based Access to HIV Prevention Services study aims to develop and evaluate a sustainable pharmacy-based model for increasing access to HIV testing and prevention services (eg, pre-exposure prophylaxis dispensing) in community pharmacy settings.

**Methods:**

We are using a hybrid type 1 effectiveness-implementation study design to evaluate the implementation and effectiveness of HIV testing and prevention services within community pharmacies, particularly among those that offer non–HIV-related screenings (eg, COVID-19, blood pressure, and cholesterol screening) and those that do not. We apply 3 well-established implementation science frameworks—the Exploration, Preparation, Implementation, Sustainment framework, the Consolidated Framework for Implementation Research, and the Systems Engineering Initiative for Patient Safety—to assess multilevel factors influencing the adoption of HIV prevention services in community pharmacy settings. This study consists of three phases: (1) a mixed methods exploration phase to identify barriers and facilitators of implementing HIV prevention services in community pharmacies, (2) a preparation phase to assess the effectiveness of two HIV training programs designed for pharmacy staff, and (3) an implementation and sustainment phase to evaluate the effectiveness and implementation of HIV prevention services in these settings.

**Results:**

The Pharmacy-Based Access to HIV Prevention Services study was funded in June 2023 by the National Institute of Mental Health (R01MH123470) and launched in September 2023. Recruitment and enrollment for the first phase, including data collection, are currently underway. We have exceeded our Phase 1 pharmacy staff survey target, with 310 participant surveys completed (goal: 300). Completion is anticipated by early 2026.

**Conclusions:**

Expanding access to HIV prevention services through community pharmacies is a promising and accessible approach to addressing social and health inequities in HIV, supporting the goal of Ending the HIV Epidemic in the United States<strong>.</strong> Implementation science, with its systematic frameworks, is essential to advancing this goal. Our study is among the first to use an implementation science approach to integrate HIV prevention services within community pharmacies in high-prevalence HIV areas.

**International Registered Report Identifier (IRRID):**

DERR1-10.2196/72283

## Introduction

### Background

Regular HIV testing and uptake of pre-exposure prophylaxis (PrEP) are the 2 most effective, yet underused HIV prevention strategies. In the United States, at least 1 in 2 Americans diagnosed with HIV is estimated to have been infected for at least 3 years [1,2], leaving a substantial window for disease progression and transmission in the community. The US Centers for Disease Control and Prevention recommends that people aged 13-64 years get tested for HIV at least once in their lifetime, and yearly for individuals at increased risk of infection (eg, people who use drugs and sexual and gender minoritized individuals) [3]. Despite this, 54% of the US population has never been tested for HIV, and only 8% have tested in the past year [4]. Similarly problematic, daily oral PrEP can prevent up to 99% of HIV infections [5], yet only 25% of those who need PrEP are currently prescribed [6,7]. Inequities in PrEP use by race are dire: in 2019, just 8% of Black people in need of PrEP received a prescription, compared to 60% of White people [8]. Just 3 years later, in 2022, PrEP uptake only increased 5% for Black people compared to a 34% increase for White people, totaling to 94% of White people who need PrEP being provided a prescription compared to 13% of Black people [8,9]. Barriers to HIV testing and PrEP uptake include limited access due to geography, medical mistrust, HIV testing stigma, and fear of a positive HIV test result [10-13].

### Community Pharmacies Are Strategic Sites for HIV Prevention

In this study, we use the term community pharmacy to refer to nonhospital, retail-based pharmacies that provide direct access to medications and basic health care services to the general public [14,15]. These include chain pharmacies, independently owned pharmacies, and those located within grocery stores or mass retailers. Beyond dispensing medications, many provide preventive and clinical services such as immunizations, health screenings, Clinical Laboratory Improvement Amendments waived testing, medication therapy management, and counseling, positioning them as important sites for public health interventions [16]. Community pharmacies are increasingly recognized as a strategic and viable solution to address documented barriers to HIV prevention services. In fact, the Centers for Disease Control and Prevention, National Institutes of Health, and other public health agencies recently declared engaging pharmacies as an integral component to Ending the HIV Epidemic (EHE) [7,14,15]. Indeed, integrating HIV prevention services in pharmacies, specifically in community pharmacy settings, could drastically expand the availability of HIV prevention services and reduce inequities in HIV for several reasons (Multimedia Appendix 1). First, about 95% of Americans live within 5 miles of a pharmacy, and most pharmacies are open beyond typical working hours (eg, 9 AM-5 PM) and on weekends [11]. Second, pharmacy staff (eg, pharmacists and pharmacy technicians) have a documented history of engaging and counseling groups at increased risk for HIV to provide HIV testing and risk reduction assessments [17-20]—even when HIV testing stigma is high [17]. Recent studies also demonstrate that pharmacy staff are especially willing to provide HIV prevention screenings [17,18,21], and that populations placed at increased risk for HIV, such as sexually minoritized men, are willing to use these services in pharmacy settings [19,20,22]. Third, data show that if pharmacies were integrated into the provision of HIV prevention services, they could increase access by over 80-fold in the US Southeast alone [23]. Lastly, integrating HIV- and non-HIV-related services can reduce stigma and increase uptake of HIV testing and prevention strategies [17]. Specifically, data show that integrating HIV testing with non-HIV services that are perceived as less stigmatizing increases pharmacy client HIV testing uptake by 42% among people who report high HIV stigma [17]. This suggests that when HIV testing is offered in a neutral, nonstigmatized setting, with other screening services that are less stigmatized (eg, cholesterol screening), it can be normalized and the fear of accepting the HIV test reduced. Recently, COVID-19 testing and vaccination have been integrated into the menu of services for many pharmacies, which opens an opportunity to integrate other infectious disease screenings [24]. Pharmacies with existing non–HIV-related screening services may be able to integrate HIV prevention services more easily than pharmacies that do not, and this may facilitate HIV testing uptake by reducing HIV exceptionalism, the fear that an HIV diagnosis is different than any other health diagnosis [17,25].

### Dissemination and Uptake of Pharmacy-Based HIV Prevention Interventions

Despite strong evidence, the dissemination and implementation of pharmacy-based HIV prevention services are extremely scarce. As of November 2024, only 14 states in the United States have passed legislation permitting pharmacists to independently provide HIV prevention services, such as prescribing PrEP [26,27]. Notably, only 1 state (ie, Arkansas) in the US South, the region most heavily impacted by HIV, has enacted such legislation [28]. The reasons for the limited adoption of similar legislation in other Southern states remain unclear. Pharmacies in the Southern region could be an accessible source for HIV prevention where other HIV prevention resources are sorely lacking [23]. Gathering evidence to explain this policy gap from local pharmacy boards that develop the protocols and regulations for pharmacy-based services is critical to implementing and scaling HIV prevention in pharmacies. On the pharmacy staff level, while many chain pharmacy staff have been able to access training to provide HIV prevention services [27], community pharmacy staff consistently report inadequate training and referral systems for positive HIV screenings as barriers to integrating HIV testing into the pharmacy work system [18,27]. Community pharmacies generally have fewer employees and may lack staff support to obtain existing training that is at least a day long [27]. Moreover, these trainings fall short of teaching pharmacy staff about oral HIV testing sample collection [27], which many community pharmacy staff want to be aware of in case their clients are unable or unwilling to perform self-HIV testing—the now standard test in many nonclinical settings [18,29].

### Implementation Science Frameworks Are Essential to Advancing Pharmacy-Based HIV Prevention Dissemination

The pharmacy work system is influenced by multiple components across various levels (eg, individual, organizational, and policy levels) [30]. In order to advance the dissemination and uptake of pharmacy-based HIV prevention services, implementation science frameworks can aid in better understanding these components and assessing how integrating HIV prevention services might drive changes within them [5]. The Exploration, Preparation, Implementation, Sustainment (EPIS) framework [18] is a widely used implementation science framework that consists of 4 well-defined phases, wherein each phase systematically addresses specific factors that can facilitate or hinder the adoption and integration of HIV prevention services within pharmacy systems. Applied to pharmacy-based HIV prevention services, EPIS is an anchoring implementation science framework that guides what is needed to advance the overall research question, rather than guide specific measures. For example, the Exploration phase would examine potential barriers and facilitators of pharmacy-based HIV prevention service integration across pharmacies, pharmacists, and pharmacy technicians. The Preparation phase would include training of pharmacy staff, increasing self-efficacy, and reducing barriers to pharmacy HIV prevention service provision. Additionally, this phase offers an opportunity to evaluate existing programs to inform and optimize implementation strategies. The Implementation phase would then test the integration of HIV prevention services in pharmacies, and the Sustainment phase would examine the multilevel influences of offering HIV prevention services.

Within EPIS, the Consolidated Framework for Implementation Research (CFIR), could guide the specific constructs and measures across each study stage [19]. CFIR posits that 5 domains influence effective implementation: intervention characteristics, outer setting, inner setting, characteristics of the individual, and process [31]. Given that community pharmacies have a unique work system, implementation science frameworks specific to the pharmacy setting are also needed to enhance our understanding of all of the pharmacy work system components [32]. Systems Engineering Initiative for Patient Safety (SEIPS) is a multilevel model previously used to guide evaluation of interrelated work systems, processes, and outcomes within the community pharmacy as implementation occurs. A number of community interventions that seek to improve client services (ie, medication therapy management and cognitive pharmaceutical services) have leveraged SEIPS, as it focuses on the design of sociotechnical systems to improve patient care [32].

The combined use of the CFIR and SEIPS models will allow both evaluation and integration of each component from a social and technical standpoint. For example, improving pharmacy-based HIV prevention access relies on implementable HIV prevention activities. In turn, these activities are completed by the pharmacists and technicians who need training and self-efficacy to implement HIV prevention screening services. This work is then supported by the pharmacy environment, which includes distal organizational structures that enable or prohibit tasks such as workflow, space, time constraints, or unrealistic compensation models [26]. Finally, pharmacy organizational factors may also underpin stigma toward HIV and HIV prevention services if these services or specific clients are singled out. Therefore, focusing on the process between work system and client factors, including acceptability and satisfaction among the pharmacy staff and clients, will be critical. Taken together, the simultaneous use of these frameworks (ie, EPIS, CFIR, and SEIPS) can shape how we understand these services and create systems that can be implemented and scaled.

### Objective

The primary goal of the Pharmacy-Based Access to HIV Prevention Services (PATH) study is to evaluate the implementation and effectiveness of HIV testing and prevention services within community pharmacies, particularly in the US Southeast. Drawing on implementation science frameworks and mixed method approaches, we aim to (1) examine policy and pharmacy staff-level barriers and facilitators of adopting HIV prevention services in community pharmacies, (2) assess existing pharmacy staff HIV prevention service trainings, and (3) test the integration of HIV prevention services in community pharmacies on effectiveness and implementation outcomes. This study uses a hybrid type 1 effectiveness-implementation design [6] that simultaneously allows us to collect data that will provide critical insight into factors that may impact PATH’s real-world implementation. In the following section, we describe the protocol for all 3 aims of PATH.

## Methods

### Study Design

The PATH study uses a mixed methods, hybrid type 1 design to test the effectiveness and evaluate the implementation of HIV prevention services in pharmacies. Two types of pharmacies will be examined (those that offer non-HIV-related screenings [eg, COVID-19, blood pressure, glucose, and cholesterol screening] and those that do not), given that integration of HIV prevention services may be different depending on the existing services offered in pharmacies. To enhance the organizational framework of the implementation and evaluation of PATH, we draw on the EPIS framework, progressing through the study in three main phases: (1) an ongoing Exploration phase with quantitative web-based surveys from community pharmacy staff and qualitative interviews with key stakeholders, including board of pharmacy members and pharmacy staff; (2) a Preparation phase to assess the effectiveness of two existing pharmacy staff centered HIV prevention training programs; and (3) a planned Implementation and Sustainment phase to test pharmacy-delivered HIV prevention services and evaluate implementation factors in 10 community pharmacies. Below, we describe the recruitment, data collection, and planned analyses for each phase.

### Conceptual Framework

**Figure 1:**
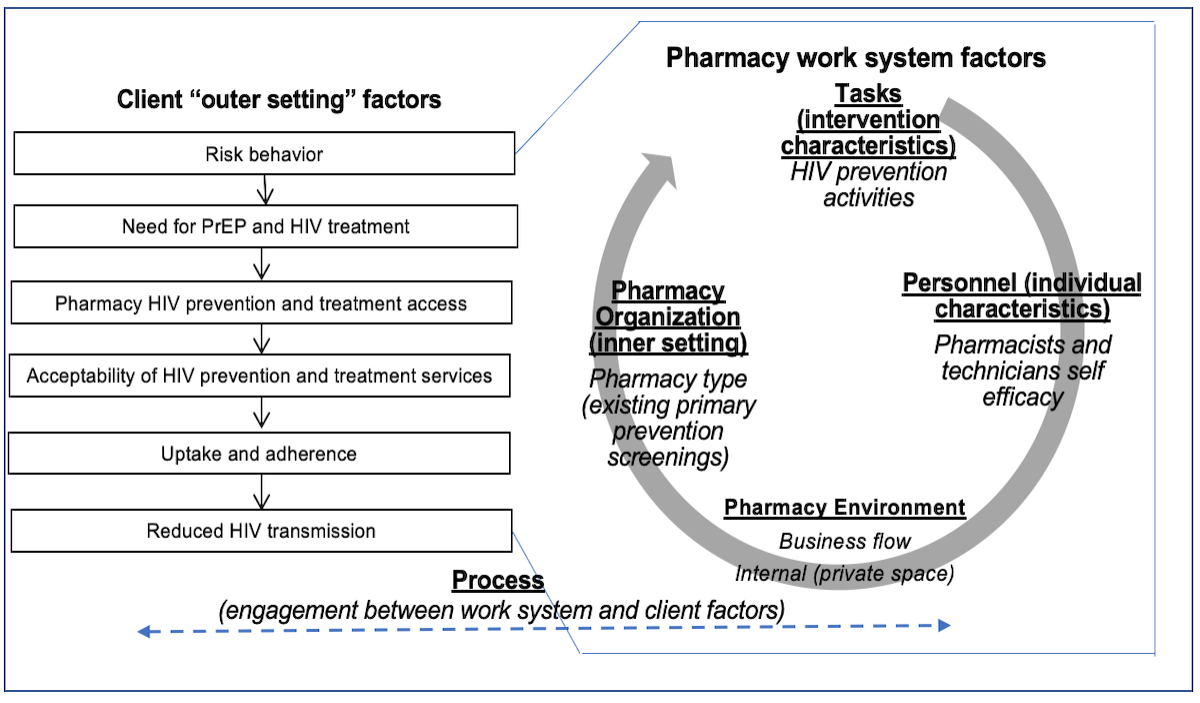
Conceptual framework for integrating HIV prevention services into pharmacies guided by the CFIR and SEIPS. CFIR: Consolidated Framework for Implementation Research; PrEP: pre-exposure prophylaxis; SEIPS: Systems Engineering Initiative for Patient Safety.

Drawing on the CFIR and SEIPS implementation science frameworks, we developed a multilevel conceptual framework for integrating HIV prevention services into pharmacies (Figure 1). The initial step to improving access relies on implementing HIV prevention activities that can be seamlessly integrated into the pharmacy setting. These activities are carried out by the pharmacists and technicians, who require proper training and self-efficacy to effectively implement HIV prevention screening services. The successful implementation of these activities is supported by the pharmacy environment, which includes organizational structures that enable or prohibit tasks such as workflow, space, time constraints, or unrealistic compensation models [11]. Finally, organizational factors within the pharmacy may contribute to stigma toward HIV and HIV prevention services, particularly if these services or specific clients are singled out. Therefore, it is critical to focus on the processes that link the work system with client factors, including acceptability and satisfaction among both pharmacy staff and clients. By addressing these elements, we aim to create a supportive environment that facilitates the successful integration of HIV prevention services into community pharmacies.

### Ethical Considerations

Ethics approval was obtained from the Emory University Institutional Review Board (STUDY00005878). All procedures involving human subjects will follow ethical principles and comply with federal regulations for the protection of human subjects. Informed consent will be obtained from all participants before participation. For the web-based survey, participants will be required to read and electronically agree to a consent form before proceeding. For qualitative interviews, verbal consent will be obtained and documented at the start of each session. All data will be deidentified before analysis. Survey data will be collected using a secure, password-protected platform. Interview transcripts will be stripped of personal identifiers during transcription. All study data will be stored on encrypted servers accessible only to research staff. Survey participants will receive a US $20 electronic gift card upon completing the survey. Stakeholders participating in interviews will receive a US $50 electronic gift card as compensation. No identifiable images of participants will be included in this paper, future study materials, or publications.

### Phase 1: Exploration Phase

The primary purpose of the Exploration phase is to establish an understanding of the policy-, pharmacy staff-, and client-level barriers and facilitators of integrating HIV prevention services into community pharmacies.

### Recruitment

#### Pharmacy Staff

To recruit pharmacy staff (pharmacists and technicians) who complete web-based surveys (n=300), we will obtain a complete list of pharmacies located in each state across EHE areas in the US Southeast from each state’s Board of Pharmacy. Pharmacy lists include the name, addresses, phone numbers, and type of pharmacy (eg, independent, chain, compounding, etc) registered to practice in each state. For states that do not make these data available (eg, AL and MS, United States), we obtained data from the Google Business application programming interface based on their location. We geocoded pharmacies to determine their locations in EHE priority jurisdictions in the Southeast [7]. We also sample from chain pharmacies within these regions. We will purposively sample participants who completed the web-based survey for 40 in-depth interviews (IDIs) with pharmacy staff. We will recontact pharmacy staff, based on pharmacy location and the presence of existing non–HIV-related screening services, to determine their willingness to participate in the IDIs.

#### Pharmacy Board Members

To recruit pharmacy board members, we will obtain a list of current board members for each state in the Southeast from their respective state Board of Pharmacy websites. We will then contact the president or chair of each board to introduce this study. Subsequently, we will request an interview with the president or chair and ask them to share introductory letters about our study with other board members to garner interest in participating. We will recruit 2 pharmacy board members from each state.

### Data Collection

#### Pharmacy Staff Survey

The 15-minute web-based survey among pharmacy staff, including pharmacists, technicians, and other pharmacy personnel, will determine the landscape of readiness for pharmacies to integrate HIV prevention services. Survey measures, informed by the CFIR and SEIPS implementation science frameworks, will include both individual and organizational-level measures of readiness such as HIV testing self-efficacy (eg, “I feel confident I can offer HIV testing to my clients”), HIV training level (eg, ever trained and training type), presence of HIV information in pharmacy, availability of a private space, time availability, HIV knowledge, attitudes and beliefs, HIV stigma (adapted from validated instruments [33]), and a checklist of health screenings provided in the pharmacy (eg, COVID-19 testing blood pressure, blood glucose and hemoglobin A_1c_ levels, and cholesterol levels). Demographic and professional characteristics such as gender, race or ethnicity, position within the pharmacy, and years of experience will also be captured to explore subgroup differences in readiness. Survey measures are described in Table 1.

**Table 1:** Assessment strategy guided by the CFIR^a^ and SEIPS^b^.

CFIR construct or SEIPS component			Description		Assessment		Specific scales from which survey items were derived or qualitative domains
**Intervention characteristics**							
	Relative advantage	Perceptions of the advantage of implementation		IDIs^c^		Open-ended questions about whether HIV testing and referral are perceived as beneficial	
	Complexity	Perceived difficulty of implementation		IDIs		Open-ended questions about the perceived difficulty of implementing HIV testing and referral	
	Tasks	HIV and non-HIV service offerings		Online surveys		Items on HIV and non-HIV related service provision from existing studies	
**Outer setting**							
	Client needs and resources	Perceived client needs; barriers and facilitators for meeting them		IDIs		Open-ended questions about HIV in the population and barriers and facilitators to pharmacy HIV testing	
	Client needs and resources	Perceived client needs; barriers and facilitators for meeting them		Surveys		Pharmacy staff HIV training level, HIV information presence in pharmacy, willingness to provide HIV testing	
	Cosmopolitanism	Knowledge of HIV organizations		IDIs		Open-ended questions on the knowledge of HIV service organizations, presence, and quality of relationships with HIV service organizations	
	External policy and incentives	Policies that could help or hinder implementation		IDIs		Open-ended questions about perceived barriers and facilitators to pharmacy HIV testing and referral implementation	
**Inner setting**							
	Structural characteristics or organization	Descriptive characteristics of pharmacy, staff, and their relation to HIV		Surveys		Checklist on non-HIV-related service provision, HIV stigma	
	Structural characteristics or organization	Descriptive characteristics of pharmacy, staff, and their relation to HIV		IDIs		Open-ended questions soliciting detailed descriptions of referral and communication processes	
	Culture	Norms, values of the specific clinic setting		IDIs		Open-ended questions about attitudes and beliefs about HIV and populations affected	
	Available resources or pharmacy environment	Descriptive characteristics of pharmacy environment		Surveys		Availability of a private space for HIV testing, time availability, and staff training	
**Individual characteristics**							
	Knowledge and beliefs	Familiarity with HIV		Surveys		Items on HIV knowledge from existing studies	
	Self-efficacy	Individual belief in their own capability to implement HIV testing in the pharmacy		Surveys		The study team will modify items from the general self-efficacy scale to measure efficacy for implementing HIV testing in pharmacies	
**Process**							
	Relative advantage	Perceptions of the advantage of implementation		IDIs		Open-ended questions about whether HIV testing and referral are perceived as beneficial	

^a^CFIR: Consolidated Framework for Implementation Research.

^b^SEIPS: Systems Engineering Initiative for Patient Safety.

^c^IDI: in-depth interview.

#### Key Stakeholder Qualitative Interviews

Interview guide measures are described in Table 1 and will cover a range of topics within 4 domains: general public health and HIV/AIDS policy, pharmacy use of health services, pharmacy-based HIV prevention services, and pharmacy-HIV organization partnerships. Informed by CFIR and SEIPS, specific IDI questions will address support for HIV prevention services, policies influencing HIV prevention service delivery, referral and monitoring in pharmacies, potential barriers to the delivery of an intervention in a pharmacy, knowledge of epidemiological data on HIV, sexual behavior, and drug use, and sustainability.

#### Key Stakeholder Compensation

Pharmacy staff who complete the 15-minute web-based survey will be sent a US $20 electronic gift card via email. Key stakeholders will be compensated with a US $50 electronic gift card for each IDI.

### Planned Analyses

#### Online Survey Data Cleaning and Analysis

Exploratory data analysis (EDA) for web-based survey data will be conducted through data editing using SAS (SAS Institute Inc) or R (R Foundation for Statistical Computing) programming. EDA will include calculations of means, medians, percentages, proportions, SDs, and skewness or kurtosis as appropriate. We will create new variables by collapsing response options when necessary. Differences in group means on continuous variables will be tested using 2-tailed *t* tests or rank tests, after interpretable normality transformations if necessary. Distributions of categorical variables will be compared between groups using chi-square tests, exact tests, or 95% CIs to guide interpretation. Group comparisons will be determined based on the outcomes of interest (eg, willingness, readiness, etc). Correlates of pharmacy HIV prevention service readiness will be examined, and relevant mediators or confounders, such as years in pharmacy practice, will be accounted for via regression modeling. The influence of outliers will be assessed, with nonparametric tests used as available and warranted. We plan to separately examine the relationship between each confounder and the outcome of interest using *t* tests or rank tests on continuous measures and exact tests of categorical values. Initial unadjusted comparisons will be made using exact tests if categorizations are used. If significant bivariate associations are found, we will incorporate these exposures as covariates using linear regression for continuous variables and logistic regression for binary variables.

#### Key Stakeholder Data Cleaning and Analysis

IDIs will be digitally recorded for verbatim transcription by a professional agency. A reflexive thematic analysis approach will be applied to all IDI data to ultimately develop a codebook that will be applied to all transcripts [8]. Analyses will integrate deductive coding using the CFIR and SEIPS frameworks as guides, along with inductive coding or analysis of emergent themes. All interviews will be coded, and all analyses will be performed by research assistants with master’s-level training in public health and qualitative methods. At each phase of data analysis, research assistants will reflexively identify any biases, values, personal factors, cultural, or other characteristics that may influence their interpretation of data. The research team will use MAXQDA Analytics Pro (VERBI Software) for data analysis.

#### Data Integration

IDIs from key stakeholders and web-based survey data will be aggregated to inform the preparation, implementation, and sustainment phases of this study. A joint display approach will be used to align quantitative findings with qualitative results. This side-by-side comparison will help determine which implementation barriers are commonly reported by both pharmacy staff and key stakeholders, and which differ between groups. Key barriers that are identified across CFIR constructs and SEIPS organizational levels will inform which aspects of the training programs require modifications to better address gaps identified during the preparation phase.

### Phase 2: Preparation Phase

The primary goal of the preparation phase is to evaluate the effectiveness of 2 existing pharmacy staff-centered HIV prevention training programs through a prepost assessment and comparative analysis. Participants from each program will complete prepost assessments to measure changes in key outcomes, including self-efficacy, HIV prevention knowledge, and readiness to prescribe PrEP. Comparative analysis will explore differences in training outcomes across the 2 programs and assess which approach more effectively addresses gaps in knowledge and practice. The anticipated components of HIV prevention training programs are detailed in Table 2.

**Table 2:** Anticipated components of HIV training modules for pharmacy staff.

Components	Description of content
Component 1	Overview of local HIV-related needs and epidemiology
Component 2	Strategies to discreetly engage customers in conversations about HIV testing
Component 3	Post-HIV testing counseling, including interpretation of HIV test results, HIV care, and discussion on risk reduction strategies
Component 4	Linkage to HIV care services, including a PrEP^a^-prescribing physician (for customers who have a nonreactive HIV test) and HIV treatment physician (for customers who have a reactive HIV test)

^a^PrEP: pre-exposure prophylaxis.

### Pharmacy Staff Training

#### Recruitment

Pharmacy staff (n=100) who completed the web-based survey in the Exploration phase will be recruited to participate in the existing pharmacy staff-centered HIV prevention training programs. If additional participants are needed, recruitment efforts will be expanded to include advertisements through local pharmacy boards, direct phone calls to pharmacies, and distribution of advertisements via mail or email. Participants will be evenly distributed between the 2 programs, with 50 individuals assigned to each HIV prevention training. Enrollment will be based on participants’ interest in enhancing their HIV prevention service delivery skills and their willingness to complete both the training and the pre- and postassessments.

#### Data Collection

To evaluate the effectiveness of 2 pharmacy-based HIV prevention training programs as a part of the preparation phase, we will administer pre- and posttraining surveys. These surveys are designed to assess changes in key outcomes, including HIV and PrEP knowledge, self-efficacy in providing HIV prevention services, and readiness to conduct HIV testing, counseling, and referral. The surveys will collect both individual-level and pharmacy-level characteristics. Individual-level variables will include gender, race, or ethnicity, position in the pharmacy, and years of experience in pharmacy practice. Pharmacy-level characteristics will include pharmacy type (eg, chain independent), geographic setting, availability of private space, and typical patient volume.

#### Planned Analyses

We will conduct both within-group and between-group analyses. Analysis of pre- and posttraining levels and comparative analyses between the 2 training programs will be conducted using variables capturing pharmacy staff self-efficacy for HIV testing, counseling, and referral, as well as HIV and PrEP knowledge. Appropriate methods for paired data, including McNemar test for binary outcomes and paired *t* or Wilcoxon signed rank tests for continuous outcomes, will be used to make inferences for each training type. For Likert scale measures, we will also consider the use of available indices for capturing change in ordinal variables based on paired data [34,35]. To comparatively assess different measures, we will report 95% CIs and use 2-group tests comparing proportions achieving positive change for binary outcomes and 2-sample *t* or rank sum tests for continuous outcomes. Univariate associations will be assessed between potential confounders and the difference between each outcome of interest pre- and posttraining, as well as the difference in differences between the training groups. When significant bivariate associations are found, we will incorporate those as covariates using linear regression for continuous outcomes and logistic regression for binary outcomes. In the comparative analysis, we will also control for pretraining differences in the outcomes across groups.

### Phase 3: Implementation and Sustainment Phase

The primary purpose of the implementation and sustainment phase is to test the efficacy of pharmacy-delivered HIV prevention services and evaluate implementation factors.

### Recruitment

#### Pharmacy Sampling, Recruitment, and Enrollment

Ten community pharmacies in Georgia will be selected to test and evaluate the pharmacy HIV prevention service implementation model. The selection will focus on community pharmacies, based on their location and client flow. Initially, we will target pharmacies identified in Phase 1 as being in the EHE priority jurisdictions in Georgia. To reach populations at the greatest risk of HIV transmission, we will choose neighborhoods with census tracts that are over 70% Black. Additionally, to ensure adequate pharmacy client recruitment in the pharmacy, we will screen pharmacies with at least 100 client visits per month. In order to achieve the target client sample, we expect 13 clients will need to be informed about this study in each pharmacy per month.

All participating pharmacies will have a private space where clients can self-administer HIV tests with complete privacy. If the pharmacy does not already have a space, additional measures will be taken to ensure participants’ privacy as needed, such as providing additional wall partitions or screens, noise machines, and noise-cancelling curtains. We will obtain consent from at least 1 pharmacist and 2 to 3 pharmacy technicians at each site to participate in this study (n=3-4 staff per pharmacy). However, we will allow participation from as many pharmacists and technicians as are willing. Given that we are recruiting from community pharmacies, which often staff fewer people, we do not expect more than 3-4 staff at each pharmacy.

#### Pharmacy Client Recruitment, Eligibility, and Enrollment

**Figure 2:**
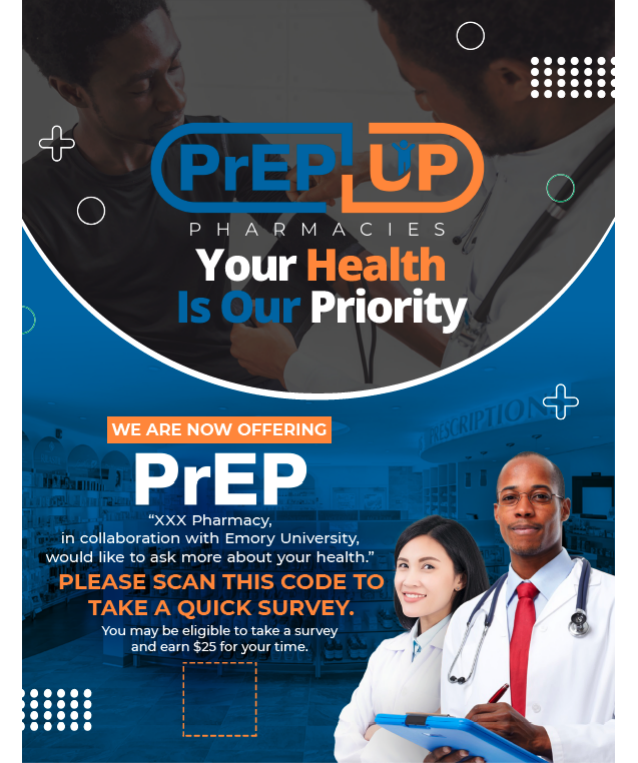
Example of a pharmacy brochure given to pharmacy clients. PrEP: pre-exposure prophylaxis. Reproduced from Crawford et al [21], which is published under a Creative Commons Attribution 4.0 International License [36].

Pharmacy staff will discreetly inform pharmacy clients about this study through brochures placed in their prescription bags and posters displayed throughout the pharmacy (refer to Figure 2 for the study flyer). Interested clients can scan a QR code to access a 10-question electronic screener survey to determine their eligibility. Participants will be eligible if they are (1) 18 years or older, (2) English-speaking, (3) have engaged in sex or drug use in the past 12 months, or (4) have never had an HIV test. Clients who complete the screener survey will receive a US $1.00 e-credit or coupon for their next pharmacy purchase. Eligible clients interested in participating will be prompted to inform a pharmacy staff member via their phone. The staff member will then offer the client free health screenings (ie, HIV self-testing as well as any other health screening already offered at the pharmacy) and inform them about the research study, which includes compensation for completing a more in-depth survey. Clients can choose to receive the health screenings or complete the survey. If they are unable to participate immediately, they will be given the option to schedule an appointment at a later date.

### HIV Prevention Service Training Evaluation

As described earlier, during the Preparation phase, pharmacy staff will participate in existing virtual training programs on HIV prevention. Following the training, research staff will schedule a 1-hour, in-person session with each pharmacy to support the integration of HIV self-testing into their services. For pharmacies without existing primary prevention screenings, the session will cover stocking self-HIV tests and configuring workflows to ensure a private area for screening. For pharmacies already offering such screenings, the session will focus on colocating HIV self-tests and advertisements alongside other primary prevention services. In all pharmacies, advertisements, including posters and brochures, will be placed strategically throughout each pharmacy to promote the availability of HIV testing (Figure 2). Designed to appeal to a broad customer base, these materials will use inclusive, nonstigmatizing language and imagery. We have tested and used similar fliers to recruit participants in other pharmacy-based studies [21]. Fliers will be positioned with other health-related literature and resources inside and outside of the pharmacy to ensure accessibility and reduce the potential for stigma by avoiding a sole focus on HIV services.

### Data Collection

#### Fidelity Assessments

To ensure consistency in the delivery of HIV testing and referral protocols by pharmacy staff, the research team will conduct periodic “mystery shopper” assessments at least once every 6 months throughout this study’s period. Pharmacy staff will be informed at enrollment and during the consent process that their services may be evaluated at various times during data collection, and that research team members will not identify themselves as part of the Emory study team during these assessments. If inconsistencies or deviations from the protocol are observed, we will inquire about these discrepancies and conduct follow-up one-on-one training sessions with pharmacy staff to address specific protocol-related concerns.

#### Pharmacy Staff Survey

Survey measures will capture implementation outcomes across the four components of SEIPS: (1) personnel, (2) the pharmacy organization, (3) the pharmacy environment, and (4) the tasks required for this study (Table 3). We will include CFIR constructs that assess barriers and facilitators to providing HIV testing and referrals across each SEIP component. To gauge the burden on pharmacy staff, we will also examine the resources required for this study. We will estimate this based on the number of clients who initiated HIV testing, HIV testing costs, time required for HIV testing counseling, PrEP, or HIV treatment referral, based on pharmacists’ time providing medication therapy management (about US $2-$3 per minute) [37,38]. These preliminary estimates will inform a formal cost-benefit analysis in the scale-up of this proposal.

**Table 3:** Variables to be collected among pharmacy staff by the SEIPS^a^ model.

Work system component	Pharmacist and technician description of variables
Personnel	Age, race or ethnicity, sex, attitudes, willingness, and perceptions of HIV prevention, PrEP^b^, and ART^c^ services, and HIV stigma
Organization	Years in practice, pharmacy workflow, average number of customers, average number of customers who fill prescriptions for STIs^d^, estimated number of customers, experience providing point of care testing, experience working with physicians, and pharmacist-physician collaborative index [39]
Pharmacy environment	Business flow, availability of internal private space for testing, and neighborhood characteristics (safety, physician access, and demographic characteristics)
Tasks	Ability to and perceptions of engaging clients about HIV testing, PrEP, and treatment, ability to set up a behavioral survey, ability to provide a biological screening kit, ability to link with a PrEP or treatment physician, ability to provide culturally appropriate information about PrEP and treatment

^a^SEIPS: Systems Engineering Initiative for Patient Safety.

^b^PrEP: pre-exposure prophylaxis.

^c^ART: antiretroviral therapy.

^d^STI: sexually transmitted infection.

#### Pharmacy Staff Retention

Participating community pharmacies will receive US $2000 per year because the activities will require more of their time and the use of a designated area of space for an unknown period each week. Each pharmacy technician (we estimate 3 technicians per pharmacy) will receive US $40 for the training session plus US $40 for each quarter of participation in this study.

#### Pharmacy Client HIV Testing

Clients in pharmacies without existing primary prevention screenings will be offered HIV testing only. Clients in pharmacies with existing primary prevention screenings will be presented with the option of doing a primary prevention health screening that includes the available primary prevention screenings as well as HIV testing. In this case, HIV testing will be highlighted as knowing your HIV status as a general health issue that also encompasses being aware of COVID-19, hypertensive, diabetic, and hypercholesterolemic status. Clients who agree to obtain an HIV test will be provided with a prepackaged kit of self-administered tests for HIV, with illustrated instructions to facilitate the oral swab. According to the processing time of the tests, the results will be apparent in no longer than 15 minutes. Clients will be asked to wait in a private area, and if they are willing, complete the 20-minute sociobehavioral survey during this time. Regardless of survey completion, they will be asked to provide their follow-up information (ie, phone number, email, address, and contact for someone who can reach them) for tracking following their HIV result. Clients who are unwilling to obtain an HIV test will be (1) counseled on the importance of HIV prevention, (2) provided with information about our community partner for future consideration, and (3) told that there is a survey that they can participate in regardless of their willingness to test.

The pharmacist or technician will interpret the results of the rapid HIV OraQuick (OraSure Technologies, Inc) test. Pharmacy staff will be trained to deliver results to the client. The pharmacy will be provided with a documentation system for maintaining the participants’ results in a locked file cabinet. Individuals who have an inconclusive HIV test will be told that their test is inconclusive and will be referred to our community partner for a confirmatory HIV test. All clients who test for HIV (regardless of reactivity) will be provided HIV posttest counseling. If a client tests positive for HIV, they will be immediately referred to our community partner for confirmatory testing and linkage to treatment services. Clients will be informed that during the PrEP referral process (counseling about the need for PrEP and identification of a nearby provider), their contact information will also be given to the PrEP-prescribing physician’s office, so the physician may also reach out to them to establish an appointment. Pharmacy clients may refuse to participate at any time, or if they have time constraints, they can schedule the HIV (and other health) screening at a later date. If they are uncomfortable completing HIV (or other health) screening in the pharmacy, but are interested in being screened, we will also provide them with a referral. We will complete a 3-month follow-up phone call with clients who obtained an HIV test to determine whether they were linked to HIV care or PrEP.

#### Pharmacy Client Survey

Clients who are eligible (based on the screener) and willing to complete the sociobehavioral survey will provide electronic informed consent via an electronic tablet. The 20-minute client surveys will collect data on demographics, sex, and drug use risk behaviors, chronic and mental health, social exposures, and feasibility, acceptability, and safety of HIV testing (Table 4).

**Table 4:** Social and behavioral survey measures.

Characteristics	Description
Demographics	Age, education level, employment, race or ethnicity, homelessness, nativity, incarceration history, health care access, health insurance coverage, housing stability, transportation access, and pharmacy practices
Sex and drug use behaviors	Sexual preferences, unprotected anal or vaginal sex, number of partners, HIV testing history, STI^a^ screening history, PrEP^b^ screening history, PrEP use, willingness to use PrEP, HIV status, partner HIV status, prescription and nonprescription drug use battery
Chronic health	History of high blood pressure, hypercholesterolemia, and diabetes
Mental health	Depression and anxiety
Social exposures	Social support, HIV stigma, and discrimination
Pharmacy service use history	Willingness and history of obtaining primary prevention screenings in pharmacies (COVID-19 testing, blood pressure, cholesterol, and glucose screening)
Feasibility	Ability to complete HIV self-screening
Acceptability	Satisfaction with where or how they were informed about HIV testing, receipt of a brochure, survey, self-screening, counseling from pharmacy staff, and PrEP or treatment referral
Safety	Perception of HIV and PrEP stigma, comfort performing self-HIV testing in the pharmacy, privacy, and confidentiality perceptions

^a^STI: sexually transmitted infection.

^b^PrEP: pre-exposure prophylaxis.

#### Pharmacy Client Compensation

Individuals who agree to complete the baseline and follow-up survey will be compensated via a US $25 electronic gift card for each complete survey.

### Planned Analyses

Upon compilation of data, new variables will be created, and variables may be collapsed as needed to explore the proportions and correlates of outcomes. EDA and data management will be conducted using SAS or R programming, as will data editing, along with calculations of descriptive statistics (eg, means, medians, percentages, proportions, SDs, and skewness or kurtosis, as appropriate). If outliers or nonstandard distributions are identified, variable transformations or standardized cut points will guide the recoding of continuous variables, following externally validated standards where available. The influence of outliers will be assessed, and medians (or rank tests) will be used if required. Primary outcomes for pharmacy staff (implementation and sustainability outcomes) and pharmacy clients (self-HIV testing uptake) will initially be compared between pharmacies offering non–HIV-related services and those that do not, using chi-square tests, exact tests, or with 95% CIs. We will examine correlates of interest for pharmacy staff (eg, years in practice and position) and clients (eg, health insurance, sexual and drug use behavior, mental health, and social exposures). Each potential confounder’s relationship to the outcome of interest will be evaluated separately using t tests or rank tests for continuous measures and exact tests for categorical measures. Those found to be univariately associated with outcomes will be included in appropriate regression models to assess the adjusted association relationship between each outcome and the presence of non–HIV-related services (yes or no). The primary analyses will account for potential clustering of individuals within pharmacies through mixed linear or nonlinear regression models. Additionally, variables with potential to modify or confound associations between outcomes and pharmacy categorization (ie, the presence or absence of HIV-related services) will be accounted for in the final model-based analyses, with appropriate interpretations.

## Results

PATH funded in June 2023. Participant recruitment and data collection began in November 2023. As of September 15, 2025, we have exceeded our Phase 1 pharmacy staff survey target, with 310 participant surveys completed (goal: 300). Data analysis for this phase is currently underway. Recruitment and data collection for Phase 1 IDI are ongoing; four IDIs have been completed at the time of this manuscript submission, with completion anticipated by early 2026.

## Discussion

### Principal Findings

PATH is a hybrid type 1 effectiveness-implementation study that focuses on integrating HIV prevention services into community pharmacies. We expect that this intervention will improve access to HIV prevention, reduce stigma around HIV in pharmacy settings, and identify the key operational challenges and systemic factors that affect the long-term success of these services. The expected findings will highlight improvements in pharmacy staff confidence, patient engagement, and the feasibility of integrating HIV prevention into everyday pharmacy practice. This study represents an important contribution to the field by integrating geospatial methods and implementation science frameworks such as CFIR and SEIPS—a model that is well-established in the industrial engineering subspecialty of human factors, but new to public health and behavioral sciences.

### Comparison to Prior Work

PATH is a promising pharmacy-based HIV prevention intervention and represents an important contribution to the field of HIV prevention service delivery for several reasons. While prior studies have shown that pharmacy-based interventions can be effective in promoting HIV prevention, they often fail to address the organizational and workflow challenges that hinder long-term sustainability. Thus, real-world effectiveness remains elusive as existing HIV prevention programs have not survived beyond the study time frame [12,13,16,38,40-43]. Many of these studies focus on the efficacy or the intervention itself but overlook the operational barriers within pharmacies, such as workflow inefficiencies, staffing challenges, and a lack of integration with other health care services. PATH aims to address these gaps by integrating theoretical frameworks used in the pharmacy discipline and integrating geospatial methods, ensuring that HIV prevention services are implemented in high-burden areas, thereby having the greatest impact. The inclusion of SEIPS provides a novel approach to examining the community pharmacy work system, focusing on human factors and organizational processes, an aspect missing from prior research. Hence, findings from this study will provide a much-needed understanding of how to incorporate and sustain HIV prevention services by characterizing specific breaks within the community pharmacy work system that promote or weaken pharmacy HIV prevention service delivery. The proposed research is expected to contribute a sustainable, pharmacy-based HIV prevention model that can be implemented in community pharmacies and serve as a foundation for expanding access to HIV prevention services, particularly among populations with limited access to existing resources. The successful development of PATH could provide evidence for the expansion to large chain pharmacies and also establish pharmacies as a venue for additional HIV prevention services, such as oral and long-acting injectable PrEP screening and dissemination, as well as PrEP checkups. By integrating HIV prevention services into pharmacies, this model has the potential to reduce strain on existing HIV health service providers, decompressing their current work systems. This outcome will be mutually beneficial for HIV public health practitioners and for community pharmacists, as provision of HIV prevention services has the potential to increase pharmacy business and revenue.

### Strengths and Limitations

PATH uses geospatial targeting to optimize service delivery and focuses on real-world implementation. The integration of both CFIR and SEIPS offers a comprehensive evaluation of the system factors influencing service delivery and sustainability. PATH is subject to 2 main limitations in its current form while we actively enroll participants. First, although we aim to recruit a sufficient sample of pharmacy staff from each state, these data will not be generalizable to all pharmacies in the US Southeast. These data will represent the context of community pharmacies in high HIV prevalence areas rather than all pharmacies. Moreover, selection bias may arise if a pharmacy or pharmacy client refuses to enter this study, potentially limiting the generalizability of the sample. Given that this study’s purpose is to develop evidence for integrating HIV prevention services into pharmacies, we are less concerned about the impact of this potential bias. Nonetheless, PATH is a necessary first step in building the evidence for integrating HIV testing into pharmacies. Second, self-report bias may arise due to surveys querying pharmacy staff’s HIV-related stigma beliefs and pharmacy clients’ sex and drug use behaviors. To mitigate this bias, we use electronic surveys, which have been shown to improve validity by minimizing invalid reports such as socially desirable answers [38], as well as ordering questions in a way to enhance recall. Lastly, logic checks in the survey will be used to prevent invalid reporting by a respondent, preventing participants from proceeding in the survey if they report inconsistent responses. Participants who consistently contradict themselves in survey responses will be excluded from these analyses.

Although this study is conducted within the context of community pharmacies in the United States, considerations around international relevance and applicability are warranted. The structure, scope, and function of community pharmacy practice vary across countries. In the United States, community pharmacies often serve as accessible sites for preventative care, where pharmacists may provide immunizations, health screenings, and counseling in addition to dispensing medications. In contrast, in many international settings, pharmacists may have limited authority to deliver clinical or preventive services due to differences in professional scope, regulatory frameworks, or reimbursement policies [15]. These differences should be considered when interpreting the generalizability of our findings. Adaptation to local policy and practice environments will be necessary for effective implementation beyond the US context.

### Conclusions

Despite these limitations, early evidence of PATH currently shows that pharmacy-based HIV prevention services in community pharmacies are implementable in the US Southeast. The PATH intervention integrated into community pharmacies provides a unique opportunity to increase access to critically needed evidence-based HIV prevention innovations, such as HIV prevention and PrEP, especially for those who are members of communities or live in neighborhoods that might be disproportionately exposed to factors that increase their HIV risk.

## Appendix

Multimedia Appendix 1 Peer review summary from PPAH - Population and Public Health Approaches to HIV/AIDS Study Section, Healthcare Delivery and Methodologies Integrated Review Group (National Institutes of Health, USA).

## Abbreviations

CFIR: Consolidated Framework for Implementation Research
EDA: exploratory data analysis
EHE: Ending the HIV Epidemic
EPIS: Exploration, Preparation, Implementation, Sustainment
IDI: in-depth interview
PATH: Pharmacy-Based Access to HIV Prevention Services
PrEP: pre-exposure prophylaxis
SEIPS: Systems Engineering Initiative for Patient Safety

## Data Availability

The data that support the findings of this study are available upon reasonable request from the corresponding author.
