# Supplementary material for: Improving Access to HIV Prevention Services in Community Pharmacies in the US Southeast: Protocol for a Hybrid Type 1 Effectiveness-Implementation Study
Source: JMIR Res Protoc. 2025 Dec 3;14:e72283. doi: 10.2196/72283 (PMC12712567; doi:10.2196/72283)
Supplement: Multimedia Appendix 1 [file resprot_v14i1e72283_app1.pdf]

SUMMARY STATEMENT

(Privileged Communication)

PROGRAM CONTACT:
Michael Stirratt
240-627-3875
stirrattm@mail.nih.gov

Release Date: 07/26/2022
Revised Date:

Application Number: 1 R01 MH132470-01

Principal Investigator

CRAWFORD, NATALIE D

Applicant Organization: EMORY UNIVERSITY

Review Group: PPAH
Population and Public Health Approaches to HIV/AIDS Study Section
AIDS - EXP. REV.

Meeting Date: 07/14/2022
Council: OCT 2022
Requested Start: 12/01/2022

RFA/PA: PA20-144
PCC: 9A-ASGA
Dual PCC: EB/JAV
Dual IC(s): DA

Project Title: Strengthening the HIV prevention continuum using pharmacies

SRG Action: Impact Score:31 Percentile:21
Next Steps: Visit https://grants.nih.gov/grants/next_steps.htm
Human Subjects: 30-Human subjects involved - Certified, no SRG concerns
Animal Subjects: 10-No live vertebrate animals involved for competing appl.
Gender: 1A-Both genders, scientifically acceptable
Minority: 1A-Minorities and non-minorities, scientifically acceptable
Age: 3A-No children included, scientifically acceptable

Project Year	Direct Costs Requested	Estimated Total Cost
1	498,000	762,086
2	498,000	762,086
3	498,000	762,086
4	498,000	762,086
5	498,000	762,086
TOTAL	2,490,000	3,810,431

ADMINISTRATIVE BUDGET NOTE: The budget shown is the requested budget and has not been adjusted to reflect any recommendations made by reviewers. If an award is planned, the costs will be calculated by Institute grants management staff based on the recommendations outlined below in the COMMITTEE BUDGET RECOMMENDATIONS section.
EARLY STAGE INVESTIGATOR
NEW INVESTIGATOR

1R01MH132470-01 CRAWFORD, NATALIE

EARLY STAGE INVESTIGATOR
NEW INVESTIGATOR

RESUME AND SUMMARY OF DISCUSSION: The proposed study will examine the effectiveness, implementation, and sustainability of integrated HIV prevention and early detection services in community pharmacies in high HIV prevalence areas. The program is designed to increase access to HIV testing, early detection, and PrEP linkage in the South; this was viewed as a significant effort in addressing ways to stem the HIV epidemic in that region of the country. The transdisciplinary investigative team is outstanding with appropriate expertise, although there was some concern regarding overlapping team contributions. The application is conceptually well grounded, and the data collection method and analysis plan are robust and supportive of the study aims. The implementation science approach is well considered and while innovation is moderate, it is appropriate for the project. There were, however, a few weaknesses that dampened enthusiasm for the project; these included the following: The applicants did not sufficiently address how the program will reduce barriers to access due to stigma; insufficient detail was provided on measures for structural and organizational characteristics of pharmacies, and how data on PrEP linkage will be collected. The fidelity plan for the Mystery Shopper component is underdeveloped; and the feasibility of conducting the study in such a large multi-state catchment area was questionable. Moreover, a few reviewers feel that the qualitative data and preliminary study do not support continuation to an RCT, viewing the aims as more developmental. Others saw this as a missed opportunity to conduct a more formal rigorous effectiveness trial. Despite, these weaknesses, which were considered minor, while a minority of the committee assessed the potential impact of the application as moderate, the great majority assessed it as ranging from high to very high.

DESCRIPTION (provided by applicant): Early HIV diagnosis is an essential component of the strategy to End the HIV Epidemic (EHE). Yet, half of the nearly 40,000 Americans who test positive for HIV every year have late (stage 3 AIDS) infection, which results in higher community transmission and poorer treatment outcomes. In the United States South, where HIV is disproportionately impacted, 50% of HIV infections are undiagnosed. Infrequent or delayed HIV testing is most often due to poor healthcare access and stigmatizing attitudes about HIV that inhibit prevention and treatment seeking. Thus, increased HIV testing access and uptake is urgently needed to normalize HIV testing and direct individuals to pre-exposure prophylaxis (PrEP) if they test HIV negative or HIV treatment if they test HIV positive. To increase HIV testing access and uptake, we propose integrating HIV prevention services within community pharmacies located in neighborhoods with high HIV prevalence. Despite promising evidence of feasibility of pharmacy-based HIV testing, it is not widely available. In fact, most local pharmacy boards lack policy guidance on HIV testing being within their scope of practice. Our work has shown that HIV testing uptake in community pharmacies can be increased if it is offered with less stigmatized non-HIV-related services (e.g., COVID19, blood pressure, glucose, and cholesterol screening). But many community pharmacy staff report inadequate training as barriers to integrating HIV testing into the pharmacy work system. Therefore, to evaluate the implementation and effectiveness of HIV testing and prevention services within community pharmacies we propose a hybrid type 1 study design that aims to 1) Examine the policy-, pharmacy staff- and client-level barriers and facilitators of adopting HIV prevention services (e.g., HIV testing, PrEP referral, HIV treatment referral) in community pharmacies, 2) Develop and implement pharmacy staff HIV prevention service (e.g., HIV testing and counseling, PrEP referral, HIV treatment referral) trainings, and 3) Test the effects of

integrating HIV prevention services in community pharmacies with existing non-HIV-related screenings versus those without on effectiveness and implementation outcomes. Guided by the Exploration, Preparation, Implementation, Sustainment framework, this study will include an exploration phase that examines the barriers and facilitators to pharmacy-based HIV prevention service provision across the US South using online surveys of community pharmacy staff (n=300), and in-depth interviews of Board of pharmacy members (n=16) and pharmacy staff (n=40). In the preparation phase, a virtual community pharmacy HIV prevention training will be developed and offered to 150 community pharmacy staff. Finally, in the implementation and sustainment phase, we will evaluate the acceptability and sustainment of the HIV prevention service delivery over 12 months in 10 community pharmacies (5 pharmacies who offer primary prevention screenings vs. 5 pharmacies who do not). Findings will be used to support scaling HIV prevention services in community pharmacies in the South where efforts to reduce HIV transmission are desperately needed to EHE.

PUBLIC HEALTH RELEVANCE: The proposed research will evaluate the implementation and effectiveness of HIV testing and prevention services in community pharmacies to increase HIV testing access and prevent delayed HIV diagnosis that results in higher community transmission and poorer treatment outcomes. Successful development of this model could establish pharmacies as a sustainable, HIV prevention source, particularly for populations with lower access to HIV prevention resources.

CRITIQUE 1

Significance: 1
Investigator(s): 1
Innovation: 5
Approach: 2
Environment: 1

Overall Impact: The overall theme – expanding the provision of preventive and early detection services from clinics to pharmacies (and other places within and outside of the traditional health system) – is powerful and promises to substantially enhance essential health systems functions in the earliest disease stages. Very rigorous implementation science design and a highly qualified transdisciplinary team of investigators. The research premise is very strong, and boosted by compelling preliminary data from the PI, and the potential for real-life impact is strong. The intervention is mildly innovative and the methods are a rigorous standard. An implementation-effectiveness randomized controlled trial would boost the causal strength of the insight from this important work.

1. Significance:

Strengths

- A powerful and highly significant central research question that drives this work: if and how can HIV prevention services be integrated into community pharmacies. New implementation knowledge and research results on this topic have major potential to substantially boost community access to HIV prevention services.
- The result of this work is potentially generalizable to places beyond pharmacies and preventive services beyond HIV.

- At the same time, the investigators argue convincingly that several other preventive services are already available in community pharmacies in the US (such as blood pressure, glucose and cholesterol screening), increasing the chances of success of integrating HIV preventive services into pharmacies.
- Strong preliminary data generated by the PI in Atlanta – strong qualitative evidence suggesting that pharmacists are strongly supporting offering HIV preventive services, but feel they require both training and infrastructural support – both of which will be addressed by the proposed research.
- Strong preliminary data supporting that diverse HIV risk populations welcome HIV preventive offerings in pharmacies and that these offerings will substantially reduce barriers to access, in particular among the most vulnerable HIV risk populations.

Weaknesses

- None.

2. Investigator(s):

Strengths

- An outstanding transdisciplinary team, with deep expertise in epidemiology, medicine, psychology, biostatistics, anthropology and sociology, and health systems and services research.
- The investigators have previous expertise in intervention research in pharmacies.

Weaknesses

- None.

3. Innovation:

Strengths

- Moderately innovative intervention – there is emerging work on the integration of pharmacies into HIV services (such as HIV testing and PrEP provision) in other contexts, such as Africa (and in some states in the US – as the investigators describe), but overall this topic is still innovative and next stages would include implementation and sustainment in real-life health systems – these real-life innovations are still outstanding and this research can contribute to their realization in Southern States in the US and globally.

Weaknesses

- The methods are rigorous but not innovative – the creative methods of design research ideation stages, in particular in the first phase of this work, the exploration phase, could add innovation to the approach, with potentially powerful results informing the design of the pharmacy intervention, over and above the evidence that more standard insight methods, such as IDI will create.
- SEIPS 3.0 is broadly a design research framework, but the methods this lens would imply are not integrated into the research approach.

4. Approach:

Strengths

- Strong implementation science design, including an explorative, preparatory, implementation, and sustainment (EPIS) research phases.
- Strong conceptual implementation science framework, integrating elements from the CFIR and the "Systems Engineering Initiative for Patient Safety" (SEIPS) frameworks, but appropriately adapting elements from these frameworks for the research at hand.

Weaknesses

- A missed opportunity for the third phase (implementation and sustainment) of this research is a rigorous causal approach to intervention implementation in an effectiveness trial – the researchers currently envision a comparison between pharmacies with vs. without existing non-HIV related services – this comparison answers a different research question from the one that seems to be the most directly relevant one for the overall scientific goal of this work: in how far pharmacies in general can reach HIV high risk populations with HIV preventive services. A stepped-wedge trial would have been one opportunity, given the training and resource requirements of the envisioned intervention.

5. Environment:

Strengths

- Excellent institutional set-up at Emory and UGA.

Weaknesses

- None.

Study Timeline:

Strengths

- Ambitious, but plausible, given the strong and long expertise of the investigators, and their specific prior experience in the setting where this research will take place.

Weaknesses

- None.

Protections for Human Subjects:

Acceptable Risks and/or Adequate Protections.

- Strong realistic safeguards in place to protect data privacy and to react in cases of potential harm to participants.

Data and Safety Monitoring Plan (Applicable for Clinical Trials Only):

Acceptable

- Overall strong DSMP -- the researchers may be somewhat too optimistic in not expecting any problems or adverse events. This research will involve participants newly learning of their HIV status, and potentially learning of impediments to routine geographic and financial access to long-term HIV treatment and prevention services, such as PrEP, which could imply psychological discomfort and trauma.

Inclusion Plans:

- Sex/Gender: Distribution justified scientifically.
- Race/Ethnicity: Distribution justified scientifically.
- For NIH-Defined Phase III trials, Plans for valid design and analysis: Not applicable.
- Inclusion/Exclusion Based on Age: Distribution justified scientifically.
- All distributions are scientifically justified -- the sample will represent the community of people at high risk of HIV in Southern states in the US.

Vertebrate Animals:
Not Applicable (No Vertebrate Animals)

Biohazards:
Not Applicable (No Biohazards)

Applications from Foreign Organizations:
Not Applicable (No Foreign Organizations)

Select Agents:
Not Applicable (No Select Agents)

Resource Sharing Plans:
Acceptable

Authentication of Key Biological and/or Chemical Resources:
Not Applicable (No Relevant Resources)

Budget and Period of Support:
Recommend as Requested

CRITIQUE 2

Significance: 4
Investigator(s): 4
Innovation: 4
Approach: 6
Environment: 2

Overall Impact: This thoughtfully crafted application is submitted by a new, early stage investigator on behalf of a team of established investigators. The implementation science oriented proposal aims to increase PrEP uptake by leveraging pharmacy settings. The academic environment is strong, but the study catchment area could be streamlined. Enthusiasm is dampened by underdeveloped aims.

1. Significance:

Strengths

- Increasing PrEP uptake via referral is a high priority.
- Background on pharmacies and a setting for preventative care is compelling.
- Inclusion of a conceptual framework.

Weaknesses

- Does not address the significant burden experienced by pharmacists and pharmacy technicians related to COVID vaccine rollout in their facilities.
- Insufficient discussion on how service integration is stigma reducing.
- In the second paragraph the investigators define community pharmacies as “chain and independent” pharmacies. In the fourth paragraph, the investigators juxtapose chain versus community pharmacies producing some confusion.

2. Investigator(s):

Strengths

- Led by a promising ESI/NI.
- Inclusion of biostatistical support.

Weaknesses

- PI has limited independent research funding.
- Large team with potential overlap in contribution.
- Multiple staff / students funded on the project in addition to the seven named Co-Investigators.

3. Innovation:

Strengths

- Use of geospatial technology is innovative.
- Leveraging SEIPS is novel.

Weaknesses

- Implementation science and pharmacy studies are not novel.

4. Approach:

Strengths

- Use of a hybrid type 1 trial and implementation science.

Weaknesses

- Aims are developmental and bring into question readiness for a full-scale trial.
- It is unclear as to why both EPIS and CFIR are needed. Either one could have been sufficient on its own, especially since the addition of bridging and innovation factors to EPIS. At the R01

level, it is unusual to begin with Exploration, since this stage often occurs in preliminary / pilot studies.

- Preliminary studies are limited and do not indicate feasibility or acceptability for the potential proposed approach.
- More details about measures included to assess “structural characteristics/organization” would enhance the rigor. For example, it is difficult to ascertain what specifically is included in the broad category of HIV stigma.
- Mystery Shopper evaluation for fidelity is under-developed.

5. Environment:

Strengths

- Academic investigator settings are excellent.

Weaknesses

- Wide pharmacy catchment area could be tightened.

Study Timeline:

Strengths

- Appropriate

Weaknesses

- Insufficient level of granularity.
- The whole of year 5 is earmarked exclusively for data analysis and dissemination.

Protections for Human Subjects:

Acceptable Risks and/or Adequate Protections

- Acceptable, but this section could be bolstered by describing the linkage to care for confirmatory testing process.

Data and Safety Monitoring Plan (Applicable for Clinical Trials Only):

Acceptable

Inclusion Plans:

- Sex/Gender: Distribution justified scientifically.
- Race/Ethnicity: Distribution justified scientifically.
- For NIH-Defined Phase III trials, Plans for valid design and analysis: Not applicable.
- Inclusion/Exclusion Based on Age: Distribution justified scientifically.
- Broad inclusion for pharmacy clients in Aim 3, but justified.

Vertebrate Animals:

Not Applicable (No Vertebrate Animals)

Biohazards:
Not Applicable (No Biohazards)

Applications from Foreign Organizations:
Not Applicable (No Foreign Organizations)

Select Agents:
Not Applicable (No Select Agents)

Resource Sharing Plans:
Acceptable

Authentication of Key Biological and/or Chemical Resources:
Not Applicable (No Relevant Resources)

Budget and Period of Support:
Budget Modifications Recommended (in amount/time)
Recommended budget modifications or possible overlap identified:

- Recommend adjusting year 5 funding level considering the planned work and evaluating the need for multiple staff / student hires to be supported on this study.

CRITIQUE 3

Significance: 2
Investigator(s): 1
Innovation: 2
Approach: 3
Environment: 1

Overall Impact: This proposal addresses a significant issue in the field of HIV by addressing longstanding barriers to HIV testing access and uptake in the U.S. South where 50% of HIV infections go undiagnosed. The overall impact of the proposal is high because the research proposed will, if successful, lay the foundation for making HIV prevention services available in community pharmacies for populations that are disconnected from HIV prevention resources. The focus on increasing access to HIV testing by delivering testing services closer to where people live is important and the methods proposed are appropriate to accomplish the stated aims. The approach has particularly strong aspects such as the comprehensiveness of the designs of specific aims 1, 2 and 3 and the use of implementation frameworks to guide data collection and analysis. Most of the weaknesses were minor. The PI has a strong track record, including conducting preliminary studies on the acceptability and feasibility of delivering HIV testing services in community pharmacies. The collaborations with Drs. Sales, Hussen, Sterk, Lyles, Sutter, and Young who bring complementary expertise in HIV prevention

implementation science, biostatistical multilevel modeling, community-based HIV prevention and PrEP and treatment linkages, and pharmacy health care delivery, workflow integration, and partnering with chain pharmacies, increase the likelihood of a successful outcome. In conclusion, despite some minor weaknesses, the potential impact of this proposal is high because it will provide evidence on whether HIV testing can be effectively delivered by community pharmacies, which will be critical to ending the HIV epidemic in the U.S. South, with broader implications for scaling up this model in other places in the U.S.

1. Significance:

Strengths

- This study is poised to address longstanding barriers to HIV testing access and uptake in the U.S. South where 50% of HIV infections go undiagnosed.
- Prior research supporting this application includes robust preliminary results demonstrating pharmacists in high HIV prevalence neighborhoods strongly support HIV prevention services in pharmacies, high HIV-risk populations support HIV prevention services in pharmacies, and HIV prevention service integration in community pharmacies has the potential to expand access. This indicates high feasibility and acceptability of the project.
- Increasing access to HIV testing and linkages to HIV prevention or HIV treatment care is important and preliminary evidence demonstrates that pharmacy-based HIV testing is feasible, yet pharmacy-based HIV testing is not widely available, particularly in the U.S. South where people face multiple barriers to accessing needed health care, including HIV testing and prevention services. This study may provide insights on barriers and facilitators that pharmacist face in delivering HIV testing, training needs of pharmacists to adopt pharmacy-based HIV testing and an approach that can be used in other settings to increase access to HIV prevention services.

Weaknesses

- No significant weaknesses identified.

2. Investigator(s):

Strengths

- The PI, Dr. Crawford, has extensive experience implementing prospective studies in pharmacy settings, including a study that is testing the impact of a randomized pharmacy intervention to provide medical and social service referrals to people who purchase syringes in pharmacies without a prescription.
- Co-Investigators have complementary experience and a history of collaboration, including on pharmacy-based HIV prevention and implementation science research, which will be important for the successful completion of the proposal.
- The partnership with NAESM, a non-profit organization based in Atlanta that provides HIV testing, STI screening, and linkage to care, as well as other health and social services.

Weaknesses

- No significant weaknesses identified.

3. Innovation:

Strengths

- The proposed research uses an implementation science approach that draws from established frameworks, including the Systems Engineering Initiative for Patient Safety multi-level model that has not been used much in the field of public health.
- Investigators propose to build on their prior geospatial expertise to optimize implementation in community pharmacies located in areas with the highest HIV burden in the proposed states. This will allow the intervention to have high impact.

Weaknesses

- The analytic methods proposed for Specific Aims 1 to 3 are not particularly innovative.

4. Approach:

Strengths

- Specific Aims 1 and 2 are well-designed to develop, test, and assess the effectiveness of the virtual pharmacy-based HIV testing training, incorporating qualitative, surveys, and pre- and post-training assessments.
- The design of the implementation and sustainment phase, i.e., the implementation pilot trial, is comprehensive and covers aspects of pharmacy staff research activities, fidelity assessments, customer logs, client behavioral surveys, and a formal cost-benefit analysis, which will be particularly important if the proposed project is successful and scaled up.

Weaknesses

- Investigator team proposed to assess the percentage of PrEP uptake at baseline and 3 months (Figure 5), however there are insufficient details of PrEP linkages and how they will collect these data from pharmacy clients.
- The proposal does not consider alternative strategies for the identification during the exploration phase (Aim 1) any major policy or other structural barriers that cannot be addressed at the individual level through training of pharmacy staff.

5. Environment:

Strengths

- The support of the Prevention and Implementation Sciences (PAIS) Core of the Center for AIDS Research, with extensive primary data collection resources, such as staff support for survey programming, data management, and follow-up tools, provide additional guarantee that appropriate resources are available to ensure successful implementation of the research project.

Weaknesses

- No significant weaknesses identified.

Study Timeline:

Strengths

- The timeline seems appropriate to address the proposed specific aims, including the implementation trial.

Weaknesses

- None identified.

Protections for Human Subjects:

Acceptable Risks and/or Adequate Protections

- Insufficient details on pharmacy client retention plans.

Data and Safety Monitoring Plan (Applicable for Clinical Trials Only):

Acceptable

- No comments.

Inclusion Plans:

- Sex/Gender: Distribution justified scientifically.
- Race/Ethnicity: Distribution justified scientifically.
- For NIH-Defined Phase III trials, Plans for valid design and analysis: Not applicable.
- Inclusion/Exclusion Based on Age: Distribution justified scientifically.
- No comments.

Vertebrate Animals:

Not Applicable (No Vertebrate Animals)

Biohazards:

Not Applicable (No Biohazards)

Applications from Foreign Organizations:

Not Applicable (No Foreign Organizations)

Select Agents:

Not Applicable (No Select Agents)

Resource Sharing Plans:

Acceptable

- Generally acceptable. However, it seems like the resource sharing plan included in the application is from a different application focused on substance use prevention and harm reduction.

Authentication of Key Biological and/or Chemical Resources:

Not Applicable (No Relevant Resources)

Budget and Period of Support:

Recommend as Requested

THE FOLLOWING SECTIONS WERE PREPARED BY THE SCIENTIFIC REVIEW OFFICER TO SUMMARIZE THE OUTCOME OF DISCUSSIONS OF THE REVIEW COMMITTEE, OR REVIEWERS' WRITTEN CRITIQUES, ON THE FOLLOWING ISSUES:

PROTECTION OF HUMAN SUBJECTS: ACCEPTABLE

INCLUSION OF WOMEN PLAN: ACCEPTABLE

INCLUSION OF MINORITIES PLAN: ACCEPTABLE

INCLUSION ACROSS THE LIFESPAN: ACCEPTABLE

COMMITTEE BUDGET RECOMMENDATIONS: The budget was recommended as requested.

Footnotes for 1 R01 MH132470-01; PI Name: CRAWFORD, NATALIE D

NIH has modified its policy regarding the receipt of resubmissions (amended applications).See Guide Notice NOT-OD-18-197 at https://grants.nih.gov/grants/guide/notice-files/NOT-OD-18-197.html. The impact/priority score is calculated after discussion of an application by averaging the overall scores (1-9) given by all voting reviewers on the committee and multiplying by 10. The criterion scores are submitted prior to the meeting by the individual reviewers assigned to an application, and are not discussed specifically at the review meeting or calculated into the overall impact score. Some applications also receive a percentile ranking. For details on the review process, see http://grants.nih.gov/grants/peer_review_process.htm#scoring.

MEETING ROSTER

Population and Public Health Approaches to HIV/AIDS Study Section
Healthcare Delivery and Methodologies Integrated Review Group
CENTER FOR SCIENTIFIC REVIEW
PPAH
07/14/2022 - 07/15/2022

Notice of NIH Policy to All Applicants: Meeting rosters are provided for information purposes only. Applicant investigators and institutional officials must not communicate directly with study section members about an application before or after the review. Failure to observe this policy will create a serious breach of integrity in the peer review process, and may lead to actions outlined in NOT-OD-22-044 at https://grants.nih.gov/grants/guide/notice-files/NOT-OD-22-044.html, including removal of the application from immediate review.

CHAIRPERSON(S)

YOUNG, APRIL MARIE, MPH, PHD
ASSOCIATE PROFESSOR
DEPARTMENT OF EPIDEMIOLOGY
COLLEGE OF PUBLIC HEALTH
UNIVERSITY OF KENTUCKY
LEXINGTON, KY 40536

MEMBERS

ABUOGI, LISA LYNN, MD
ASSOCIATE PROFESSOR
DEPARTMENT OF PEDIATRICS
SCHOOL OF MEDICINE
UNIVERSITY OF COLORADO, DENVER
AURORA, CO 80045

AMIRKHANIAN, YURI A, PHD
PROFESSOR
DEPARTMENT OF PSYCHIATRY AND BEHAVIORAL MEDICINE
CENTER FOR AIDS INTERVENTION RESEARCH
MEDICAL COLLEGE OF WISCONSIN
MILWAUKEE, WI 53202

ARONSON, IAN DAVID, PHD *
ASSOCIATE RESEARCH SCIENTIST
CENTER FOR DRUG USE AND HIV HCV RESEARCH
SCHOOL OF GLOBAL PUBLIC HEALTH
NEW YORK UNIVERSITY
NEW YORK, NY 10003

BARNIGHAUSEN, TILL, MD
PROFESSOR AND DIRECTOR
HEIDELBERG INSTITUTE OF GLOBAL HEALTH
UNIVERSITY OF HEIDELBERG
HEIDLEBURG, GERMANY 69120
GERMANY

BLANK, MICHAEL B, PHD *
PROFESSOR
DEPARTMENT OF PSYCHIATRY
PERELMAN SCHOOL OF MEDICINE
UNIVERSITY OF PENNSYLVANIA
PHILADELPHIA, PA 19104

BOEKELOO, BRADLEY O, PHD *
PROFESSOR
DEPARTMENT OF BEHAVIORAL AND COMMUNITY HEALTH
SCHOOL OF PUBLIC HEALTH
UNIVERSITY OF MARYLAND
COLLEGE PARK, MD 20742

BUDHWANI, HENNA, PHD *
ASSOCIATE PROFESSOR
DEPARTMENT OF HEALTH CARE ORGANIZATION
AND POLICY
SCHOOL OF PUBLIC HEALTH
THE UNIVERSITY OF ALABAMA AT BIRMINGHAM
BIRMINGHAM, AL 35924

DARBES, LYNAE A, PHD
ASSOCIATE PROFESSOR
DEPARTMENT OF HEALTH BEHAVIOR
AND BIOLOGICAL SCIENCES
SCHOOL OF NURSING
UNIVERSITY OF MICHIGAN
ANN ARBOR, MI 48109

DEGRUTTOLA, VICTOR GERARD, DSC *
PROFESSOR
DEPARTMENT OF BIOSTATISTICS
SCHOOL OF PUBLIC HEALTH
HARVARD UNIVERSITY
BOSTON, MA 02115

EDLAND, STEVEN DYAL, PHD *
PROFESSOR
DEPARTMENT OF FAMILY MEDICINE AND
PUBLIC HEALTH
UNIVERSITY OF CALIFORNIA, SAN DIEGO
LA JOLLA, CA 92093

FOX, MATTHEW ALEXANDER PEASE, DSC, MPH *
PROFESSOR
DEPARTMENT OF EPIDEMIOLOGY
SCHOOL OF PUBLIC HEALTH
BOSTON UNIVERSITY
BOSTON, MA 02118

FUJIMOTO, KAYO, PHD
DISTINGUISHED PROFESSOR
DEPARTMENT OF HEALTH PROMOTION
AND BEHAVIORAL SCIENCES
SCHOOL OF PUBLIC HEALTH
UNIVERSITY OF TEXAS HEALTH SCIENCE CENTER
HOUSTON, TX 77030

GOEDEL, WILLIAM C, PHD *
ASSISTANT PROFESSOR
DEPARTMENT OF EPIDEMIOLOGY
SCHOOL OF PUBLIC HEALTH
BROWN UNIVERSITY
PROVIDENCE, RI 02912

GOPALAPPA, CHAITRA, PHD *
ASSOCIATE PROFESSOR
DEPARTMENT OF MECHANICAL
AND INDUSTRIAL ENGINEERING
COLLEGE OF ENGINEERING
UNIVERSITY OF MASSACHUSETTS, AMHERST
AMHERST, MA 01003

HAHN, JUDITH ALISSA, PHD *
PROFESSOR
DEPARTMENT OF MEDICINE
SAN FRANCISCO GENERAL HOSPITAL
UNIVERSITY OF CALIFORNIA, SAN FRANCISCO
SAN FRANCISCO, CA 94143

HECKMAN, TIMOTHY GLENN, PHD *
PROFESSOR AND ASSOCIATE DEAN
DEPARTMENT OF HEALTH PROMOTION AND BEHAVIOR
COLLEGE OF PUBLIC HEALTH
UNIVERSITY OF GEORGIA
ATHENS, GA 30602

HERBECK, JOSHUA T, PHD *
ASSISTANT PROFESSOR
DEPARTMENT OF GLOBAL HEALTH
UNIVERSITY OF WASHINGTON
SEATTLE, WA 98195

JENNESS, SAMUEL, MPH, PHD *
ASSOCIATE PROFESSOR
DEPARTMENT OF EPIDEMIOLOGY
ROLLINS SCHOOL OF PUBLIC HEALTH
EMORY UNIVERSITY
ATLANTA, GA 30030

KERSHAW, TRACE S, PHD *
PROFESSOR
CENTER FOR INTERDISCIPLINARY RESEARCH ON AIDS
DEPARTMENT OF EPIDEMIOLOGY
SCHOOL OF PUBLIC HEALTH
YALE UNIVERSITY
NEW HAVEN, CT 06510

LAU, BRYAN, PHD *
PROFESSOR
DEPARTMENT OF EPIDEMIOLOGY
BLOOMBERG SCHOOL OF PUBLIC HEALTH
JOHNS HOPKINS UNIVERSITY SCHOOL OF MEDICINE
BALTIMORE, MD 21205

LEITNER, THOMAS K, PHD *
STAFF SCIENTIST
THEORETICAL BIOLOGY AND BIOPHYSICS GROUP
LOS ALAMOS NATIONAL LABORATORY
LOS ALAMOS, NM 87545

MCMAHON, JAMES M, PHD
ASSOCIATE PROFESSOR AND ENDOWED CHAIR
SCHOOL OF NURSING
UNIVERSITY OF ROCHESTER MEDICAL CENTER
ROCHESTER, NY 14642

MEEK, ERIN, DRPH, MPH *
SENIOR RESEARCH SCIENTIST
AIDS OFFICE
SAN FRANCISCO DEPARTMENT OF PUBLIC HEALTH
SAN FRANCISCO, CA 94102

NASH, DENIS, MPH, PHD *
PROFESSOR
DEPARTMENT OF EPIDEMIOLOGY AND BIOSTATISTICS
SCHOOL OF PUBLIC HEALTH
CITY UNIVERSITY OF NEW YORK
NEW YORK, NY 10035

NIJHAWAN, ANK ELISABETH, MD, MPH *
ASSOCIATE PROFESSOR
INTERNAL MEDICINE, DIVISION OF INFECTIOUS DISEASES
UT SOUTHWESTERN MEDICAL CENTER
DALLAS, TX 75390

OUTLAW, ANGULIQUE Y, PHD *
ASSOCIATE PROFESSOR
DEPARTMENT OF FAMILY MEDICINE AND
PUBLIC HEALTH SCIENCES
SCHOOL OF MEDICINE
WAYNE STATE UNIVERSITY
DETROIT, MI 48202

PHO, MAI TUYET, MD, MPH
ASSOCIATE PROFESSOR
DEPARTMENT OF MEDICINE
SECTION OF INFECTIOUS DISEASES AND GLOBAL HEALTH
UNIVERSITY OF CHICAGO MEDICAL CENTER
CHICAGO, IL 60637

RAMIREZ (KITCHEN), CHRISTINA MICHELLE, PHD *
ASSOCIATE PROFESSOR
DEPARTMENT OF BIOSTATISTICS
SCHOOL OF PUBLIC HEALTH
UNIVERSITY OF CALIFORNIA, LOS ANGELES
LOS ANGELES, CA 90095

SALEEM, HANEEFA TASLEEM, MPH, PHD *
ASSISTANT PROFESSOR
BLOOMBERG SCHOOL OF PUBLIC HEATH
JOHNS HOPKINS UNIVERSITY
BALTIMORE, MD 21205

SALEMI, MARCO, PHD
PROFESSOR
DEPARTMENT OF PATHOLOGY, IMMUNOLOGY,
AND LABORATORY MEDICINE
COLLEGE OF MEDICINE
UNIVERSITY OF FLORIDA
GAINESVILLE, FL 32610

SEAL, DAVID W, PHD *
PROFESSOR
DEPARTMENT OF GLOBAL COMMUNITY HEALTH
AND BEHAVIORAL SCIENCES
SCHOOL OF PUBLIC HEALTH AND TROPICAL MEDICINE
TULANE UNIVERSITY
NEW ORLEANS, LA 70112

VARDAVAS, RAFFAELE, PHD *
MATHEMATICIAN
FACULTY PARDEE RAND GRADUATE SCHOOL
RAND CORPORATION
SANTA MONICA, CA 90407

WITTE, SUSAN S, PHD
PROFESSOR
SCHOOL OF SOCIAL WORK
COLUMBIA UNIVERSITY
NEW YORK, NY 10027

MAIL REVIEWER(S)

GOLIN, CAROL E, MD
PROFESSOR
DEPARTMENT OF HEALTH BEHAVIOR AND
HEALTH EDUCATION
UNIVERSITY OF NORTH CAROLINA
CHAPEL HILL, NC 27599

SCIENTIFIC REVIEW OFFICER

GUERRIER, JOSE H, PHD
SCIENTIFIC REVIEW OFFICER
CENTER FOR SCIENTIFIC REVIEW
NATIONAL INSTITUTES OF HEALTH
BETHESDA, MD 20892

* Temporary Member. For grant applications, temporary members may participate in the entire meeting or may review only selected applications as needed.

Consultants are required to absent themselves from the room during the review of any application if their presence would constitute or appear to constitute a conflict of interest.
